# Prefabricated zirconia crowns versus alternative full-coverage restorations for primary anterior teeth: a systematic review of clinical outcomes

**DOI:** 10.1007/s40368-025-01142-2

**Published:** 2025-12-09

**Authors:** A. S. Khalil, R. S. Alrehaili

**Affiliations:** 1 Department of Orthodontics, Faculty of Dentistry, Alexandria University, Alexandria, Egypt; 2 Department of Orthodontics, College of Dentistry, Arab Academy for Science, Technology and Maritime Transport (AASTMT), Alamein, Egypt; 3 Private Sector, Medina, Saudi Arabia

**Keywords:** Aesthetic crowns, Deciduous anterior teeth, Composite strip crowns, Pre-veneered stainless steel crowns, Early childhood caries, Paediatric dentistry

## Abstract

**Background:**

Prefabricated zirconia crowns have gained popularity as an alternative to traditional options such as composite strip crowns and pre-veneered stainless steel crowns. However, their comparative clinical effectiveness remains uncertain. This systematic review aimed to evaluate the clinical outcomes of prefabricated zirconia crowns (ZCs) for restoring primary anterior teeth compared to alternative full-coverage restorations.

**Methods:**

This systematic review was conducted in alignment with PRISMA guidelines. A comprehensive search of five databases, PubMed, Cochrane Library, Embase, Scopus, and Web of Science, was performed to identify eligible randomised controlled trials (RCTs) addressing the PICO question: “How does the clinical performance of prefabricated ZCs compare to alternative full-coverage restorations in primary anterior teeth?” The Cochrane Risk of Bias 2 (RoB 2) tool was used to assess the methodological quality of the included studies. Due to the heterogeneity of studies, a narrative synthesis of the findings was carried out.

**Results:**

Out of 993 records identified, seven RCTs involving 326 participants were included. ZC exhibited superior retention, with survival rates of 95–100% at 12–18 months, compared with 46–79% for composite strip crowns (CSC) and 100% for pre-veneered stainless steel crowns. Gingival health was significantly better with ZC. Bleeding scores ranged from 0 to 40% in ZC vs. 20–67% in CSC, and plaque-free surfaces were reported in 80–100% of ZC vs. 40–80% of CSC. Aesthetic outcomes favoured ZC, with colour match 98–100% vs. 44–70% for CSC. Parental satisfaction was highest for ZC, whilst secondary caries was absent in all ZC groups. RoB 2 assessment judged six studies as having “some concerns” and one as “high risk.”

**Conclusion:**

Current evidence suggests that prefabricated zirconia crowns show promising clinical performance for restoring primary anterior teeth, with advantages in retention, gingival health, and aesthetics. Nonetheless, concerns regarding enamel wear and limited long-term data highlight the need for further high-quality trials.

**Supplementary Information:**

The online version contains supplementary material available at 10.1007/s40368-025-01142-2.

## Background

Oral health is a fundamental component of a child’s overall well-being and development. Amongst the most widespread paediatric dental conditions is early childhood caries (ECC), a multifactorial disease that can develop soon after the eruption of the first primary tooth (Han et al. 2025; Paglia 2025; Patel et al. 2025). ECC is defined by the American Academy of Pediatric Dentistry (AAPD) as the presence of one or more decayed, missing due to caries, or filled tooth surfaces in any primary tooth in a child under the age of six (Irvine et al. 2011). If not managed appropriately, ECC can lead to pain, infection, difficulty in eating and speaking, and long-term impacts on general health and psychosocial development (Chou et al. 2025; Feldens et al. 2010; Li et al. 2020). The premature loss of maxillary anterior teeth in particular can negatively impact a child’s facial aesthetics, phonetics, and psychosocial health (Gupta et al. 2019).

Restoring anterior primary teeth affected by ECC presents significant clinical challenges, particularly in young children who may be uncooperative or require treatment under sedation or general anaesthesia (Zhang et al. 2023). The treatment requires careful consideration of aesthetics, durability, gingival compatibility, and patient comfort. Over the years, numerous full-coverage restorative options have been developed to address these needs. These include composite strip crowns (CSCs), pre-veneered stainless steel crowns (PVSSCs), and more recently, prefabricated zirconia crowns (ZCs). Composite strip crowns have long been the most commonly used aesthetic restorations for anterior primary teeth due to their translucency, adaptability, and affordability (Kupietzky et al. 2003; Suresh and Gunasekaran 2024; Waggoner 2002). They offer good initial colour matching, are easy to trim, and blend well with natural dentition (Ram and Fuks 2006). However, CSCs are highly technique-sensitive. Successful placement requires moisture control, which is often difficult to achieve in young children without sedation or general anaesthesia (Psaltis and Kupietzky 2008). This limitation can result in compromised bonding, colour changes, marginal leakage, and ultimately, reduced crown longevity (Basra et al. 2024).

To address these limitations, aesthetic preformed crowns were introduced. Amongst them, ZCs have gained popularity due to their superior mechanical properties and aesthetic advantages (Alrashdi et al. 2022; Planells del Pozo and Fuks 2014; Shrestha et al. 2020; Soleimani et al. 2020). These crowns are biocompatible, highly durable, and resistant to staining and plaque accumulation (Chaudhary et al. 2025). Their smooth glazed surfaces are less prone to bacterial colonisation, potentially improving gingival health (Pei and Chen 2023). Unlike CSCs, ZCs do not require bonding procedures sensitive to contamination. However, ZCs do require more aggressive tooth preparation and are less adaptable in cases of crowding or minimal remaining tooth structure (Clark et al. 2016; Lee 2018). Their relatively high cost and limited shade availability also pose constraints (Pate et al. 2021).

Despite their increasing use in paediatric dentistry, there remains a lack of consensus regarding the optimal crown type for restoring carious anterior teeth. Most clinical evidence stems from small-scale trials or observational studies with limited follow-up. Additionally, direct comparisons between ZCs and other crown types are sparse, and the literature varies in terms of evaluation methods and outcomes. Whilst several studies have highlighted the high retention rates, aesthetic satisfaction, and gingival health associated with ZCs (Bani-Hani et al. 2025; Chowdhary et al. 2021; Murali et al. 2022; Patnana et al. 2022), others caution that their effectiveness depends heavily on case selection and preparation technique (Chaudhary et al. 2025; Clark et al. 2016; Lee 2018). Given the clinical implications of crown selection in paediatric patients, a thorough appraisal of the existing evidence is warranted. A clear synthesis of existing evidence will enable clinicians to make more informed material choices that optimise treatment efficiency, aesthetic outcomes, and long-term success for primary anterior teeth. This is especially important in minimising retreatments under general anaesthesia and improving parental satisfaction—two factors that significantly influence paediatric dental care quality.

Given the above considerations, the objective of this systematic review is to evaluate the clinical outcomes of prefabricated ZCs in comparison to alternative full-coverage restorations for restoring primary anterior teeth. The review focuses on multiple outcome domains including retention, gingival health, plaque accumulation, aesthetic satisfaction, secondary caries, enamel wear on opposing teeth, and overall parental or patient satisfaction.

## Methods

### Protocol, registration, and focussed question

This systematic review was developed in accordance with the Preferred Reporting Items for Systematic Reviews and Meta-Analyses (PRISMA) guidelines. The protocol was registered on the Open Science Framework database (https://osf.io/uacxp/). The purpose was to systematically assess the available evidence in relation to the focussed research question: “How do the clinical outcomes of prefabricated ZCs compare to those of alternative full-coverage restorative options for primary anterior teeth?”.

### Eligibility criteria

The inclusion criteria followed the PICOS framework:

- **Population (P):** Paediatric patients with carious or defective primary anterior teeth requiring full-coverage restoration.
- **Intervention (I):** Prefabricated zirconia crowns.
- **Comparison (C):** Alternative anterior crown types, including CSC, PVSSC, or other aesthetic restorations.
- **Outcome (O):** clinical success parameters such as crown retention, gingival health, plaque accumulation, aesthetics, secondary caries, enamel wear on opposing teeth, and parental/child satisfaction.
- **Study Design (S):** randomised controlled clinical trials (RCTs) with a minimum follow-up of 6 months.

### Inclusion and exclusion criteria

Eligible studies were peer-reviewed RCTs published in English that compared the clinical performance of prefabricated ZCs against other full-coverage restorative options in primary anterior teeth. Studies were excluded if they: (a) focussed exclusively on primary posterior teeth, (b) did not include prefabricated zirconia crowns, (c) were not RCTs, (d) lacked comparative clinical data relevant to the review objective, or (e) included teeth with developmental defects.

### Information sources and search strategy

A systematic literature search was conducted in five databases: PubMed, Cochrane Library, Embase, Scopus, and Web of Science. The search included all articles from database inception until August 18, 2025, and incorporated both MeSH terms and free-text keywords related to ZCs and paediatric dental restorations. A manual search of the bibliographies of included papers was conducted to identify further eligible publications (Supplementary file 1).

### Study selection and assessment

Two reviewers (A.S.K and R.S.A) independently performed the screening and selection processes. Any disagreements were resolved through discussion.

### Data extraction

Data were extracted using a standardised form and included: study characteristics (author, year, country), sample demographics, intervention and comparator details, follow-up duration, outcome measures, and key findings.

### Quality assessment

The methodological quality of included RCTs was evaluated using the Cochrane Risk of Bias 2 (RoB 2) tool, covering five core domains: randomisation process, deviations from intended interventions, missing outcome data, outcome measurement, and selection of reported results. Each domain was rated as low risk, some concerns, or high risk. An overall risk of bias judgement was then assigned following the RoB 2 guidelines. Studies were classified as low risk if all domains were rated low, high risk if at least one domain was rated high, and some concerns if at least one domain raised concerns but none were rated high (Sterne et al. 2019).

## Results

### Study selection

**Fig. 1:**
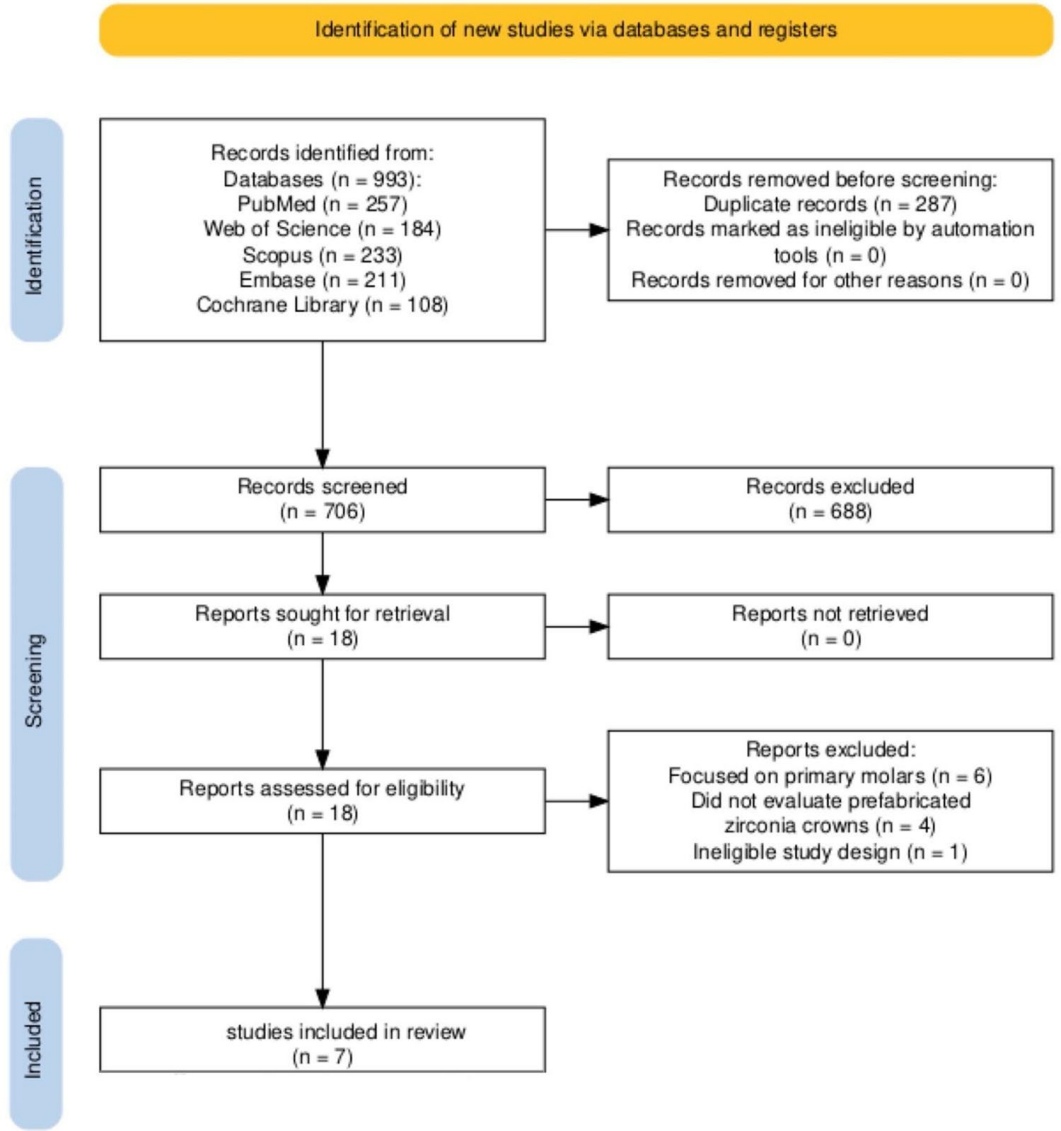
PRISMA 2020 flowchart

The study selection process is outlined in Fig. 1. A total of 993 records were initially identified through electronic database searches. After removing 287 duplicate entries, 706 records remained for title and abstract screening. Of these, 688 articles were excluded for not meeting the inclusion criteria based on relevance and study type. Subsequently, 18 full-text articles were assessed for eligibility. Following full-text evaluation, 11 studies were excluded for the following reasons: six focussed on posterior teeth, four did not evaluate prefabricated zirconia crowns, and one employed an ineligible study design (Supplementary file 2). Ultimately, seven randomised controlled trials met all eligibility criteria and were included in the qualitative synthesis (Alaki et al. 2020; Gill et al. 2020; Labbé et al. 2023; Misgar et al. 2022; Özdemir et al. 2022; Ravada et al. 2022; Vaghela et al. 2021). These studies compared prefabricated ZCs with other full-coverage restorative approaches for primary anterior teeth, focussing on clinical performance outcomes.

### Study characteristics

The included seven randomised controlled trials investigated the clinical outcomes of prefabricated ZCs for the restoration of primary anterior teeth. The trials were published between 2020 and 2023 and were conducted in various international settings, including the United States (Gill et al. 2020), Canada (Labbé et al. 2023), Saudi Arabia (Alaki et al. 2020), Turkey (Özdemir et al. 2022), and India (Misgar et al. 2022; Ravada et al. 2022; Vaghela et al. 2021). All studies were carried out in paediatric dental departments of academic institutions or university-affiliated hospitals.

Each trial applied a two-arm parallel-group design, where prefabricated ZCs were compared against alternative full-coverage restorative options. The comparator crowns included CSCs (Alaki et al. 2020; Gill et al. 2020; Labbé et al. 2023; Özdemir et al. 2022; Vaghela et al. 2021), resin-based crowns (Ravada et al. 2022), PVSSCs (Gill et al. 2020), and Luxa crowns (Misgar et al. 2022). In total, 326 participants were involved across all studies**.** Sample sizes ranged from 25 (Özdemir et al. 2022) to 100 children (Ravada et al. 2022), with participant ages predominantly falling between 3 and 6 years (Alaki et al. 2020; Ravada et al. 2022; Vaghela et al. 2021)**.** Gender distribution was reported in four of the included trials, comprising 84 males (51%) and 81 females (49%) (Alaki et al. 2020; Gill et al. 2020; Labbé et al. 2023; Özdemir et al. 2022), although a few did not specify exact breakdowns (Misgar et al. 2022; Ravada et al. 2022; Vaghela et al. 2021). The clinical outcomes assessed varied across studies but primarily focussed on crown retention, gingival health, plaque accumulation, marginal adaptation, colour match, secondary caries, enamel wear, and both caregiver and child satisfaction (Alaki et al. 2020; Gill et al. 2020; Labbé et al. 2023; Misgar et al. 2022; Özdemir et al. 2022; Ravada et al. 2022; Vaghela et al. 2021). Evaluation methods included standardised assessment tools such as the United States Public Health Service (USPHS) criteria (Alaki et al. 2020; Gill et al. 2020; Özdemir et al. 2022; Vaghela et al. 2021), Silness and Löe plaque and gingival indices (Alaki et al. 2020; Özdemir et al. 2022; Vaghela et al. 2021), visual scoring systems (Misgar et al. 2022; Ravada et al. 2022), and Likert-style satisfaction scales (Gill et al. 2020; Vaghela et al. 2021). Follow-up extended from 6 to 18 months, with some studies incorporating multiple time-point assessments (Labbé et al. 2023; Misgar et al. 2022; Özdemir et al. 2022; Ravada et al. 2022; Vaghela et al. 2021) (Table 1).

**Table 1:** Study population, interventions, and outcomes of included studies

Author and year	Country	Participants (Gender)	Age (mean/range)	Intervention Groups	Comparator Groups	Outcomes Evaluated	Lesion extent/surfaces	Isolation method	Anaesthesia	Outcome Assessment Method	Follow-up Period
Gill et al. (2020)	United States	*N* = 49 (M: 26, F: 23)	3.4 years	ZC (*n* = 61 teeth)	CSC (*n* = 18 teeth); PVSSC (*n* = 56 teeth)	Crown retention, marginal adaptation, gingival health, colour match, parental satisfaction	Full coverage for carious/fractured	Rubber dam	GA	USPHS criteria, Likert-scale questionnaires	12 months
Alaki et al. (2020)	Saudi Arabia	*N* = 32 (M: 12, F: 20)	4.43 ± 0.7 years	ZC (*n* = 60 teeth)	CSC (*n* = 60 teeth)	Gingival bleeding, plaque index, secondary caries, crown failure, enamel wear	≥ 2 surfaces involved	Rubber dam for CSC |	Local with nitrous oxide	Silness and Löe plaque index, USPHS, Smith and Knight Tooth Wear Index	12 months
Vaghela et al. (2021)	India	*N* = 31 (gender not specified)	3–6 years	ZC (*n* = 47 teeth)	CSC (*n* = 55 teeth)	Clinical performance, parental satisfaction, child preference	Carious incisors	Not reported	Not reported	USPHS, Löe and Silness index, Likert and smiley face scales	Baseline, 3, and 9 months
Misgar et al. (2022)	India	*N* = 30 (gender not specified)	4–9 years	ZC (*n* = 10 teeth)	Luxa Crowns (*n* = 10 teeth); Resin Strip Crowns (*n* = 10 teeth)	Gingival health, secondary caries				WHO probe, visual inspection, chi-square analysis	3, 6, and 9 months
Özdemir et al. (2022)	Turkey	*N* = 25 (M: 13, F: 12)	4.5 ± 0.83 years	ZC (*n* = 43 teeth)	CSC (*n* = 43 teeth); Caries-free control group (*n* = 43 untreated incisors, for plaque comparisons only)	Crown retention, gingival health, plaque, colour match, pulpal health	ECC incisors	Split rubber dam	LA	USPHS criteria, clinical scores	1, 6, 12, and 18 months
Ravada et al. (2022)	India	*N* = 100 (gender not specified)	3–6 years	ZC (*n* = 50 teeth)	CSC (*n* = 50 teeth)	Gingival health, plaque, restoration failure, secondary caries, enamel wear	ECC incisors	Not reported	|Not reported	Clinical inspection, visual assessment	3, 6, and 12 months
Labbé et al. (2023)	Canada	*N* = 59 (M: 33, F: 26) ZC: M: 12, F: 14 CSC: M: 21, F: 12	33.0 ± 8.8 mo (ZC); 32.3 ± 7.0 mo (CSC)	ZC (*n* = 76 teeth)	CSC (*n* = 101 teeth)	Restoration integrity, pulp therapy, feasibility	Full coverage for carious/fractured	Rubber dam	GA	Standardised photographs, blinded review, GEE model	6 and 12 months

N, number of participants; M, male; F, female; SD, standard deviation; ZC, zirconia crown; CSC, composite strip crown; PVSSC, pre-veneered stainless steel crown; GA, general anaesthesia; LA, Local anaesthesia; USPHS, United States Public Health Service criteria; WHO, World Health Organization; GEE, generalised estimating equation

### Quality assessment

A summary of the risk of bias judgments is presented in Table 2. All seven included studies were judged as having either "low risk" or "some concerns" in most domains. The most commonly observed concerns were related to allocation concealment and selection of the reported results. In the domain of randomisation, most studies employed appropriate random sequence generation, although details regarding concealment were often insufficiently reported. Blinding of participants and caregivers posed another common source of concern. Due to the visible differences between zirconia and comparator crown types, full blinding of operators was often unfeasible. However, several studies ensured that participants, typically young children under anaesthesia or sedation, were unlikely to be aware of treatment allocation (Gill et al. 2020; Vaghela et al. 2021). Nevertheless, operator awareness was frequently noted and led to a judgement of "some concerns" in this domain. Ultimately, six of the seven studies were rated as having "some concerns" in risk of bias, primarily due to methodological limitations related to blinding and reporting transparency (Alaki et al. 2020; Gill et al. 2020; Labbé et al. 2023; Misgar et al. 2022; Özdemir et al. 2022; Vaghela et al. 2021). One study was rated as having a high risk of bias due to insufficient detail on randomisation, lack of assessor blinding, and a higher potential for detection bias (Ravada et al. 2022) (Supplementary file 3).

**Table 2:** Risk of bias judgments assessed using Risk of Bias 2 (RoB 2) tool

Author and Year	Randomisation	Deviations from Intervention	Missing Outcome Data	Measurement of Outcomes	Selection of Reported Result	Overall Risk of Bias
Gill et al. (2020)	Some concerns	Some concerns	Low risk	Low risk	Some concerns	**Some concerns**
Alaki et al. (2020)	Low risk	Low risk	Low risk	Low risk	Some concerns	**Some concerns**
Vaghela et al. (2021)	Some concerns	Some concerns	Low risk	Low risk	Some concerns	**Some concerns**
Misgar et al. (2022)	Some concerns	Some concerns	Low risk	Low risk	Some concerns	**Some concerns**
Özdemir et al. (2022)	Some concerns	Some concerns	Low risk	Low risk	Some concerns	**Some concerns**
Ravada et al. (2022)	Some concerns	Some concerns	Low risk	Some concerns	Some concerns	**High risk**
Labbé et al. (2023)	Low risk	Some concerns	Low risk	Low risk	Low risk	**Some concerns**

### Results of individual studies and main findings

The included randomised controlled trials assessed a range of clinical outcomes for ZC compared with alternative full-coverage restorations (CSC, PVSSC, and Luxa crowns). A summary of results is presented in Table 3.

**Table 3:** Key findings from included studies

Author and year	Retention / Failure	Gingival health	Plaque	Aesthetics (colour/match)	Secondary caries	Opposing enamel wear	Parent/child satisfaction	Principal conclusion
Gill et al. (2020)	CSC 79% vs PVSSC 100% vs ZC 95% (*P* = 0.002). Marginal adaptation and facing integrity also favoured ZC/PVSSC (*P* < 0.001)	ZC/PVSSC better; CSC worst (bleeding NS)	–	Colour match: CSC 44% vs PVSSC 100% vs ZC 98% (*P* < 0.001)	None	–	87% very satisfied; dissatisfaction higher with CSC and PVSSC colour (*P* = 0.005)	ZC and PVSSC showed superior retention and aesthetics compared with CSC
Alaki et al. (2020)	12 mo: ZC 1.7% complete loss vs CSC 38.3% total (*P* < 0.001). 6 mo: ZC 1.7% vs SC 13.4% (*P* = 0.024)	3 mo: CSC 66.7% vs ZC 40% (*P* = 0.006). 6 mo: CSC 46.7% vs ZC 0% (*P* < 0.001). Both 0% at 12 mo	Plaque-free: ZC consistently higher (3 mo 33.3% vs 6.7%; 6 mo 79.7% vs 40%; 12 mo 100% vs 80%). All *P* ≤ 0.001	–	12 mo: ZC 0% vs CSC 6.7% (NS, *P* = 0.135)	12 mo: wear in 11.7% opposing ZC vs 0% CSC (*P* = 0.020)	–	ZC showed better survival, gingival, and plaque outcomes, but greater opposing enamel wear
Vaghela et al. (2021)	9 mo intact: CSC 80.4% vs ZC 97.8% (*P* = 0.002)	9 mo inflammation-free: SC 82.4% vs ZC 100% (*P* = 0.003)	–	Colour match at 9 mo: SC 70.6% vs ZC 100% (*P* = 0.001)	None	No wear; margins closed 100% both groups	Parents less satisfied with SC for colour (*P* = 0.042) and durability (*P* = 0.002). Children preferred ZC	ZC demonstrated higher retention, aesthetics, and satisfaction compared with SC
Misgar et al. (2022)	–	Bleeding: 3 mo CSC 40% vs ZC 20% vs Luxa 20% (*P* < 0.05). 6 mo SC 20% vs ZC/Luxa 0%. All 0% at 9 mo	–	–	9 mo: SC 40% vs ZC 0% vs Luxa 0% (*P*≈0.04). All 0% at 3 & 6 mo	–	–	ZC and Luxa had no caries and better gingival response; SC showed higher caries at 9 mo
Özdemir et al. (2022)	Retention: ZC 100% vs SC 77.8% at 18 mo (*P* = 0.023). Failures: SC 42.9% minor loss vs ZC 0% (*P* < 0.001)	Gingival index: significant group & time effects (*P* < 0.001); interaction NS	ZC had lower plaque than SC and controls at all recalls (all *P* < 0.001)	Colour stability favoured ZC at 12 mo (*P* = 0.005) and 18 mo (*P* = 0.008)	None	–	–	ZC performed better in retention, plaque, and colour stability; pulpal outcomes similar to CSC
Ravada et al. (2022)	CSC had significantly more failures at 6 & 12 mo (*χ*^2^, *P* < 0.05). ZC losses mainly trauma-related	ZC had less bleeding at 3 & 6 mo (*P* < 0.05)	ZC had lower plaque at 3 & 6 mo (*P* < 0.05)	–	12 mo: no difference (NS)	Opposing wear in 10 ZC teeth vs 0 SC	–	ZC favoured for gingival health, plaque, and survival; higher opposing enamel wear noted
Labbé et al. (2023)	Intact crowns: 6 mo ZC 85.5% vs SC 57.4% (OR 4.2, 95% CI 1.3–13.3). 12 mo ZC 81.4% vs SC 46.5% (OR 4.0, 95% CI 1.2–13.0)	–	–	–	Caries included within failure outcomes; not separately reported	–	–	ZC had approximately fourfold higher odds of intact survival; pulp therapy rates were similar

ZC, zirconia crown; CSC, composite strip crown; PVSSC, pre-veneered stainless steel crown; Luxa, Luxa crown; OR, odds ratio; CI, confidence interval; NS, not significant; mo, month; *χ*^2^, chi-square test

### Retention

ZC consistently demonstrated higher retention rates. Across studies, intact retention ranged from 95 to 100% at 12 months, compared with 46–79% for CSC, and 100% for PVSSC. Two trials reported odds ratios for survival, showing ZC had approximately fourfold higher odds of intact crowns at 6–12 months compared with CSC (OR 4.0–4.2, *P* ≤ 0.02) (Labbé et al. 2023; Özdemir et al. 2022).

### Gingival health

Five RCTs reported gingival indices or bleeding. ZC showed significantly lower bleeding scores than CSC at early recalls. Bleeding scores were significantly lower for ZC compared with CSC at 3–6 months, ranging from 0 to 40% in ZC versus 20–67% in CSC (*P* < 0.05) (Alaki et al. 2020; Misgar et al. 2022; Ravada et al. 2022). At later follow-ups, ZC crowns often showed 0% bleeding, whilst CSC continued to present inflammation (Vaghela et al. 2021).

### Plaque accumulation

Four RCTs assessed plaque indices. ZC was associated with significantly lower plaque accumulation than CSC across all follow-ups. At 6 months, plaque-free crowns were recorded in 79.7% of ZC versus 40% of CSC (*P* < 0.001). One study reported plaque-free surfaces in 100% of ZC compared with 80% of CSC at 12 months (*P* = 0.001) (Alaki et al. 2020).

### esthetics

Three RCTs compared aesthetic outcomes. ZC consistently had significantly higher colour match scores (98–100%) at 9–12 months, whereas CSC showed markedly lower ratings (as low as 44–70%, *P* < 0.001).

### Secondary caries

Three RCTs reported on secondary caries. ZC and Luxa groups showed no secondary caries across 6–12 months, whilst CSC showed caries rates of 6.7–40%, with significance in some trials but non-significant in others.

### Enamel wear on opposing teeth

Two RCTs reported wear of opposing dentition. ZC was associated with some enamel wear: one study observed 11.7% opposing wear at 12 months, which was significantly higher than the CSC group (*P* = 0.020) (Alaki et al. 2020), and another reported wear in 10 opposing teeth (Ravada et al. 2022). No wear was reported with CSC.

### Pulpal health

One RCT evaluated pulpal health outcomes and reported no significant differences between ZC and CSC in terms of pulpal response throughout the 18-month follow-up, with both groups showing stable pulpal status and no pathological changes on clinical or radiographic assessment (*P* > 0.05) (Özdemir et al. 2022).

### Parental and child satisfaction

Three RCTs reported satisfaction outcomes. Parents consistently rated ZC higher for aesthetics and durability, with dissatisfaction significantly greater for CSC and PVSSC. In one study, 87% of parents were very satisfied overall, but dissatisfaction was concentrated in CSC (63%) and PVSSC (37%) groups, representing a significant difference (*P* = 0.005) (Gill et al. 2020). Children also preferred ZC over CSC in aesthetic evaluations (Vaghela et al. 2021).

### Quantitative synthesis of the results

Differences were evident in the types of comparator restorations, outcome measures, and follow-up durations. This level of heterogeneity made it inappropriate to combine the results in a quantitative meta-analysis.

## Discussion

Restoration of decayed or compromised primary anterior teeth presents a multifactorial challenge, particularly in young children where aesthetics, durability, and gingival health are key concerns. This systematic review synthesised evidence from seven RCTs comparing prefabricated ZCs with alternative full-coverage restorations. Across all trials, ZC demonstrated superior outcomes in retention, gingival health, plaque accumulation, and aesthetics, with no recurrent caries reported in ZC groups in most studies. CSC had higher rates of failure, caries, and poorer aesthetics. These findings are consistent with previous clinical and observational studies that have reported favourable performance of ZCs in paediatric anterior restorations (Alhissan and Pani 2021; Yanover et al. 2021a; Yanover et al. 2021b). However, a consistent concern was opposing enamel wear associated with ZC.

Retention and material stability are critical for long-term clinical success. ZCs achieved high retention rates, often reaching or approaching 100% at follow-up points ranging from 6 to 18 months (Özdemir et al. 2022; Ravada et al. 2022; Vaghela et al. 2021). In contrast, CSCs showed notably lower retention, with failures often attributed to their technique sensitivity and dependency on moisture control and the need for sufficient tooth structure for bonding (Gill et al. 2020; Misgar et al. 2022; Özdemir et al. 2022). One trial reported a retention rate of only 77.8% for CSCs compared to 100% for ZCs at 18 months (Özdemir et al. 2022), which aligns with previous findings of Gill et al. (2020), who reported 95% retention for ZCs versus 79% for CSCs at 12 months. Longer-term retrospective data reinforce these findings. Kupietzky et al. (2003) retrospectively evaluated 112 CSCs in 40 children and found an 88% retention rate after approximately 18 months, with none of the restorations being completely lost and only 12% showing partial resin loss. In an extended follow-up, Kupietzky et al. (2005) examined 145 CSCs and observed a slightly reduced retention rate of 80% after an average of 31.3 months, with partial loss of resin in 20% of cases but no complete failures. These findings confirm that CSCs can perform acceptably over time but remain vulnerable to marginal breakdown and chipping. Earlier research emphasised the clinical reliability of ZCs in young children, even in high-risk cases requiring pulp therapy or extensive coronal coverage (Alrashdi 2024; Rocha et al. 2021).

It is worth noting, however, that not all evidence favours zirconia in every context. A recent 2.5-year retrospective cohort study found significantly higher failure rates in ZCs compared to stainless steel crowns following pulpotomy in primary molars (Brenner et al. 2025). Importantly, these failures were primarily linked to pulpotomy outcomes rather than crown integrity, as the major complications included abscess and fistula. Whilst these findings pertain to posterior teeth and different clinical scenarios, they emphasise the need for cautious extrapolation and longer-term anterior-specific trials.

CSC are also prone to chipping and material loss, likely due to bonding failure or marginal leakage (Özdemir et al. 2022; Misgar et al. 2022). Conversely, ZCs, with their monolithic design and use of resin-modified glass ionomer cement (RMGIC), provide more stable outcomes and minimise marginal failures (Vaghela et al. 2021; Ravada et al. 2022). These advantages reflect zirconia’s high flexural strength, fracture resistance, and reduced susceptibility to microleakage compared to composite restorations (Waggoner and Cohen 1995; Alonso and Caserio 2012).

ZCs consistently outperformed CSCs in preserving gingival health. Their highly polished, biocompatible surfaces limit bacterial adhesion and biofilm formation, which likely explains the lower bleeding and inflammation scores observed clinically (Özdemir et al. 2022). Alaki et al. (2020) demonstrated significantly reduced plaque accumulation and gingival bleeding in ZC-treated teeth compared to CSCs after 12 months. Further supporting this, Mathew et al. (2020) found that *Streptococcus mutans* adhered less to posterior zirconia surfaces compared to stainless steel crowns, with lower plaque scores and gingival inflammation sustained across a 12-month follow-up period. Immunological and microbiological findings also echo this trend. Saharia et al. (2024) reported that children restored with posterior ZCs exhibited lower interleukin-6 (IL-6) levels and reduced *Lactobacillus casei* counts compared to those with stainless steel crowns, indicating better periodontal tissue response and lower microbial colonisation. These findings highlight zirconia’s favourable interaction with the surrounding gingival environment. Moreover, Abdelhafez and Dhar (2025) compared posterior stainless steel crowns, ZCs, and Bioflx crowns and found that zirconia showed superior results in terms of plaque control and gingival index at both 6- and 12-month assessments. Nonetheless, some variability in results was observed in the present review. One study reported that both ZC and CSC groups exhibited gingival inflammation that was higher than the caries-free control group, likely due to poor oral hygiene and the increased mobility of exfoliating incisors (Özdemir et al. 2022)**.** The authors also noted that limited brushing compliance, especially during the COVID-19 pandemic, might have contributed to poorer outcomes, irrespective of crown material. These challenges highlight the importance of parental support and oral hygiene instruction following crown placement, regardless of the restoration used.

Aesthetics was a central concern across most trials, with ZCs consistently outperforming CSCs in both parental and child satisfaction measures (Gill et al. 2020; Labbé et al. 2023; Vaghela et al. 2021). Whilst CSCs offer good initial aesthetics with customisable shades, their colour stability deteriorates over time due to surface roughness, staining, and marginal breakdown. Vaghela et al. (2021) reported that 29.4% of CSCs exhibited shade mismatch at 9 months, whereas ZCs maintained 100% colour stability. These findings reinforce conclusions from earlier studies reporting that discoloration and chipping are major limitations of CSC in long-term use (Alonso and Caserio 2012). These aesthetic limitations were also documented by Kupietzky et al. (2003), who noted that teeth receiving pulpotomy prior to CSC placement frequently displayed significant colour mismatch, especially when obturated with iodoform-based pastes. Furthermore, in a follow-up study of caregiver perspectives, Kupietzky and Waggoner (2004) found that although colour mismatch was the most common aesthetic complaint amongst parents, it did not significantly affect overall satisfaction. Interestingly, the study revealed a statistically significant relationship between perceived durability and overall satisfaction, suggesting that function and longevity may outweigh cosmetic imperfections in caregiver perception. Children’s perspectives further reinforce the advantage of zirconia. Surveys using Likert scales and smiley face evaluations revealed that children expressed greater satisfaction with ZCs, often citing durability and aesthetics as reasons (Murali et al. 2022; Özdemir et al. 2022; Vaghela et al. 2021). For instance, 92.8% of children rated ZCs as "very happy" at 9 months compared to only 66.7% for CSCs (Özdemir et al. 2022). These results echo prior research that emphasises the increasing aesthetic awareness of both caregivers and paediatric patients, and the important psychosocial role of anterior dental appearance in early childhood (Lee 2002).

Enamel wear on opposing dentition emerged as a concern in some of the included trials. Ravada et al. (2022) reported wear in 10 opposing teeth restored with ZCs at 12 months, whereas no wear was recorded in the CSCs. Similarly, Alaki et al. (2020) found enamel wear in 11.7% of opposing teeth after 12 months in the ZC group, compared to none in the CSC group. Such concerns are consistent with earlier in vitro research showing that zirconia, whilst biocompatible, may exhibit abrasive effects on antagonist teeth over time (DeLong et al. 1989).

However, such wear was not a universal finding. Trials with shorter follow-ups reported no differences. Vaghela et al. (2021) and Özdemir et al. (2022) both found no evidence of opposing enamel wear up to 9–18 months. Given the short functional lifespan of primary dentition, the clinical impact of enamel wear remains uncertain, but careful occlusal adjustment may help minimise risk.

Regarding pulpal health, no significant differences were found between ZCs and CSCs in the majority of the studies. Although ZCs generally require more extensive tooth preparation, which may theoretically increase pulpal stress, none of the included studies reported adverse pulpal outcomes directly attributable to ZC use. Radiographic follow-up in multiple trials confirmed that both crown types preserved pulpal vitality over time (Labbé et al. 2023; Misgar et al. 2022)**.** These findings are supported by prior clinical evidence indicating that when proper restorative technique is followed, ZCs do not compromise biological safety, even in endodontically treated teeth (Walia et al. 2014).

The findings of this review indicated that ZC demonstrated superior outcomes in retention, gingival health, plaque accumulation, and aesthetics, with no secondary caries reported in ZC groups in most studies. A small but consistent concern was opposing enamel wear associated with ZC. Nevertheless, considerations such as cost, preparation requirements, and follow-up duration remain important factors in clinical decision-making.

### Strengths, limitations, clinical implications, and future perspectives

This systematic review possesses several notable strengths that enhance the validity and relevance of its findings. It is focussed exclusively on RCTs with a minimum follow-up of 3 months, thereby ensuring a high level of evidence and minimising the risk of selection and performance biases inherent in observational studies. Moreover, the review was conducted in accordance with PRISMA guidelines and registered prospectively, which promotes methodological transparency and reproducibility (Supplementary file 4). However, several limitations in the current body of evidence must be acknowledged. First, although all studies were RCTs, many were rated as having “some concerns” in at least one domain of the RoB2 tool, particularly due to insufficient reporting of allocation concealment and the inability to blind operators or outcome assessors. Second, heterogeneity existed across studies in terms of comparator crown types, evaluation criteria, and outcome measurement tools. For example, some trials used subjective parental satisfaction surveys or non-standardised visual scoring (Ravada et al. 2022; Vaghela et al. 2021), whilst others employed more objective indices such as USPHS criteria or Silness and Löe plaque and gingival indices (Alaki et al. 2020; Gill et al. 2020; Özdemir et al. 2022). Third, most studies included only healthy, cooperative children treated in university settings, potentially limiting the generalisability of findings to broader paediatric populations, including those with behavioural challenges or systemic health conditions. Additionally, the review was restricted to studies published in English, which may have introduced language bias and led to the exclusion of relevant trials published in other languages, whilst the omission of grey literature raises the possibility of publication bias. Finally, due to heterogeneity in outcome definitions, follow-up durations, and comparator groups, a meta-analysis could not be performed, limiting the ability to generate pooled effect estimates.

The results of this systematic review have several important implications for clinical practice and future research. From a clinical standpoint, prefabricated ZCs demonstrate consistently superior performance to traditional full-coverage restorations, particularly in terms of crown retention, gingival health, aesthetics, and caregiver satisfaction. These findings support the routine use of ZCs in paediatric dental settings, especially for patients and families with high aesthetic expectations. However, the decision to use ZCs must also take into account potential trade-offs, such as increased cost, the need for greater tooth reduction, and the possibility of enamel wear on opposing teeth. Future research should aim to address existing gaps in the evidence. Longitudinal studies with extended follow-up periods are needed to assess long-term crown survival, impact on occlusion, and wear on opposing dentition. Additionally, standardisation of outcome measures, use of validated satisfaction scales, and inclusion of broader paediatric populations, such as uncooperative children or those with special healthcare needs, will enhance the external validity and generalisability of findings.

## Conclusions

Prefabricated zirconia crowns appear to offer superior retention, gingival health, and aesthetics compared to alternative full-coverage restorations for primary anterior teeth. However, some concerns remain regarding potential enamel wear on opposing dentition. Further long-term trials are warranted to confirm their long-term performance and validate these findings.

## Supplementary Information

Below is the link to the electronic supplementary material.

Supplementary file1 (DOCX 16 KB)

Supplementary file2 (DOCX 19 KB)

Supplementary file3 (DOCX 46 KB)

Supplementary file4 (DOCX 33 KB)

## Data Availability

No datasets were generated or analysed during the current study.

## Abbreviations

ZC: Zirconia crown
CSC: Composite strip crown
PVSSC: Pre-veneered stainless steel crown
USPHS: United States Public Health Service
RCT: Randomised controlled trial
PRISMA: Preferred reporting items for systematic reviews and meta-analyses
RoB 2: Risk of bias 2 tool
ECC: Early childhood caries

## References

[CR1] Abdelhafez A, Dhar V. Comparative clinical performance of stainless steel, zirconia, and Bioflx crowns in primary molars: a randomized controlled trial. BMC Oral Health. 2025;25(1):585. 40251538 10.1186/s12903-025-05869-8 PMC12007260

[CR2] Alaki SM, Abdulhadi BS, AbdElBaki MA, Alamoudi NM. Comparing zirconia to anterior strip crowns in primary anterior teeth in children: a randomized clinical trial. BMC Oral Health BioMed Central. 2020;20(1):1–11. 10.1186/s12903-020-01305-1 PMC7654025 33167954

[CR3] Alhissan AS, Pani SC. Factors influencing the survival of preformed zirconia crowns in children treated under general anesthesia. Int J Dent. 2021;5:1–5. 10.1155/2021/5515383 PMC8012123 33833801

[CR4] Alonso V, Caserio M. A clinical study of direct composite full-coverage crowns: long-term results. Oper Dent. 2012;37(4):432–41. 22335302 10.2341/11-229-S

[CR5] Alrashdi M. Survival analysis of prefabricated zirconia crowns with and without pulpotomy in primary teeth: a retrospective cohort study. Children. 2024;11(11):1402. 39594977 10.3390/children11111402 PMC11592978

[CR6] Alrashdi M, Ardoin J, Liu JA. Zirconia crowns for children: a systematic review. Int J Paediatr Dent. 2022;32(1):66–81. 33772904 10.1111/ipd.12793

[CR7] Bani-Hani T, Al-Fodeh RS, Al-Wahadni AM, Abu-Alhaija ES, Al-Hakam M. An in-vitro investigation into the fracture resistance of prefabricated and custom-made zirconia crowns for permanent molars in children. Dent J. 2025;13(2):113–9. 10.3390/dj13020064 PMC11854477 39996938

[CR8] Basra AS, Manik K, Khetan RR, Sawarbandhe PA. Smile renewal: incisor restoration using the strip crown technique. Cureus. 2024;6(9):e123. 10.7759/cureus.63716 PMC11298765 39105013

[CR9] Brenner I, Abdin M, Schmoeckel J. Pediatric preformed zirconium oxide crowns vs. preformed metal crowns after pulpotomy in primary molars: a practice-based retrospective 2.5 year cohort study. Healthcare. 2025;13(7):751. 40218049 10.3390/healthcare13070751 PMC11988542

[CR10] Chaudhary UC, Singh M, Srivastava D, Hugar S, Singh V, Singh N, et al. Comparative study on the durability of stainless steel crowns vs. zirconia crowns for pediatric use. J Pharm Bioallied Sci. 2025;17(Suppl 2):S1631. 40655616 10.4103/jpbs.jpbs_199_25 PMC12244821

[CR11] Chou YC, Cheng FS, Weng SH, Hu HY. Risk of severe early childhood caries over time in low-income preschoolers. JDR Clin Transl Res. 2025;10(2):146–56. 10.1177/23800844241279266 39359105

[CR12] Chowdhary R, Ajayakumar LP, Chowdhary N, Reddy VR. Use of restorative full crowns made with zirconia in children: a systematic review. Int J Clin Pediatr Dent. 2021;13(5):551–8. 10.5005/jp-journals-10005-1822 PMC7887175 33623346

[CR13] Clark L, Wells MH, Harris EF, Lou J. Comparison of amount of primary tooth reduction required for anterior and posterior zirconia and stainless steel crowns. Pediatr Dent. 2016;38(1):42–6. 26892214

[CR14] DeLong R, Sasik C, Pintado MR, Douglas WH. The wear of enamel when opposed by ceramic systems. Dent Mater. 1989;5(4):266–71. 2638270 10.1016/0109-5641(89)90073-0

[CR15] Feldens CA, Giugliani ERJ, Vigo Á, Vítolo MR. Early feeding practices and severe early childhood caries in four-year-old children from southern Brazil: a birth cohort study. Caries Res. 2010;44(5):445–52. 20838043 10.1159/000319898

[CR16] Gill A, Garcia M, Won An S, Scott J, Seminario AL. Clinical comparison of three esthetic full-coverage restorations in primary maxillary incisors at 12 months. Pediatr Dent. 2020;42(5):367–72. 33087221

[CR17] Gupta T, Sadana G, Rai HK. Effect of esthetic defects in anterior teeth on the emotional and social well-being of children: a survey. Int J Clin Pediatr Dent. 2019;12(3):229–32. 31708620 10.5005/jp-journals-10005-1628 PMC6811947

[CR18] Han S-Y, Chang C-L, Wang Y-L, Wang C-S, Lee W-J, Vo TTT, et al. A narrative review on advancing pediatric oral health: comprehensive strategies for the prevention and management of dental challenges in children. Children (Basel, Switzerland). 2025;12(3):14–9. 10.3390/children12030286 PMC11941194 40150569

[CR19] Irvine J, Holve S, Krol D, Schroth R. Early childhood caries in Indigenous communities: a joint statement with the American academy of pediatrics. Paediatr Child Health. 2011;16(6):351–64. 22654547 10.1093/pch/16.6.351 PMC3328230

[CR20] Kupietzky A, Waggoner WF. Parental satisfaction with bonded resin composite strip crowns for primary incisors. Pediatr Dent. 2004;26(4):337–40. 15344627

[CR21] Kupietzky A, Waggoner WF, Galea J. The clinical and radiographic success of bonded resin composite strip crowns for primary incisors. Pediatr Dent. 2003;25(6):577–81. 14733473

[CR22] Kupietzky A, Waggoner WE, Galea J. Long-term photographic and radiographic assessment of bonded resin composite strip crowns for primary incisors: results after 3 years. Pediatr Dent. 2005;27(3):221–5. 16173227

[CR23] Labbé S, Garisto GA, Barrett EJ, Casas MJ. A comparison of primary maxillary incisor zirconia and composite resin strip crowns: a one-year feasibility study. Pediatr Dent. 2023;45(2):113–6. 37106539

[CR24] Lee JK. Restoration of primary anterior teeth: review of the literature. Pediatr Dent. 2002;24(5):506–10. 12412966

[CR25] Lee J-H. Guided tooth preparation for a pediatric zirconia crown. J Am Dent Assoc. 2018;149(3):202-208.e2. 29395008 10.1016/j.adaj.2017.08.048

[CR26] Li J, Fan W, Zhou Y, Wu L, Liu W, Huang S. The status and associated factors of early childhood caries among 3- to 5-year-old children in Guangdong, Southern China: a provincial cross-sectional survey. BMC Oral Health. 2020;20(1):265. 32977784 10.1186/s12903-020-01253-w PMC7517683

[CR27] Mathew MG, Samuel SR, Soni AJ, Roopa KB. Evaluation of adhesion of Streptococcus mutans, plaque accumulation on zirconia and stainless steel crowns, and surrounding gingival inflammation in primary molars: randomized controlled trial. Clin Oral Investig. 2020;24(9):3275–80. 31955271 10.1007/s00784-020-03204-9

[CR28] Misgar BA, Goyal V, Rani N. Comparative evaluation between zirconia, luxa andstrip crowns: a randomised controlled trial. J Adv Med Dental Sci Res. 2022;22(12):1462–70.

[CR29] Murali G, Mungara J, Vijayakumar P, Keerthi T, Kothimbakkam SSK, Priya AS. Clinical evaluation of pediatric posterior zirconia and stainless steel crowns: a comparative study. Int J Clin Pediatr Dent. 2022;15(1):9–14. 35528490 10.5005/jp-journals-10005-2125 PMC9016913

[CR30] Özdemir M, Unverdi GE, Geduk N, Ballikaya E, Cehreli ZC. Clinical comparison of preformed zirconia and composite strip crowns in primary maxillary incisors: 18-month results of a prospective, randomized trial. Pediatr Dent. 2022;44(6):416–22. 36947757

[CR31] Paglia L. Early childhood caries: a family-centred disease. Eur J Paediatr Dent. 2025;26(2):87. 40470895 10.23804/ejpd.2025.26.02.01

[CR32] Pate JD, Wells MH, Morrow BR, Ragain JC, Garcia-Godoy F. Color stability of prefabricated pediatric zirconia crowns following sterilization. Pediatr Dent. 2021;43(1):50–6. 33662251

[CR33] Patel NS, Mehta M, Fu Y, Desai V, Lala HS, Parikh H, et al. A review of early childhood caries: risk factors, management, and policy recommendations. Cureus. 2025;17(5):e83767. 40486439 10.7759/cureus.83767 PMC12145511

[CR34] Patnana AK, Chugh VK, Chugh A, Vanga NRV, Kumar P. Effectiveness of zirconia crowns compared with stainless steel crowns in primary posterior teeth rehabilitation. J Am Dent Assoc. 2022;153(2):158-166.e5. 35086644 10.1016/j.adaj.2021.08.005

[CR35] Pei S-L, Chen M-H. Comparison of periodontal health of primary teeth restored with zirconia and stainless steel crowns: a systemic review and meta-analysis. J Formos Med Assoc. 2023;122(2):148–56. 36180321 10.1016/j.jfma.2022.08.015

[CR36] Planells del Pozo P, Fuks A. Zirconia crowns - an esthetic and resistant restorative alternative for ECC affected primary teeth. J Clin Pediatr Dent. 2014;38(3):193–5. 25095311 10.17796/jcpd.38.3.0255q84jt2851311

[CR37] Psaltis GL, Kupietzky A. A simplified isolation technique for preparation and placement of resin composite strip crowns. Pediatr Dent. 2008;30(5):436–8. 18942605

[CR38] Ram D, Fuks AB. Clinical performance of resin-bonded composite strip crowns in primary incisors: a retrospective study. Int J Paediatr Dent. 2006;16(1):49–54. 16364093 10.1111/j.1365-263X.2006.00680.x

[CR39] Ravada SUS, Nara A, Gavarraju DN, Arya J, Kapur I, Begani S. Comparison of efficiency of different crowns in primary children an original research. Int J Health Sci. 2022;6(1):13197–202.

[CR40] Rocha MCM, Inácio GC, Taira TM, Delgado RZR, Maciel SM, Frítola M. Zirconia crowns as an esthetic alternative for oral rehabilitation in pediatric dentistry: a review. Pediatr Dent J. 2021;31(3):224–34.

[CR41] Saharia NP, Malik M, Jhingan P, Gulati N, Mathur S. Assessment of interleukin-6 levels and *Lactobacillus casei* counts in pediatric stainless steel and zirconia crowns: a comparative study. Int J Clin Pediatr Dent. 2024;17(4):395–403. 39144182 10.5005/jp-journals-10005-2813 PMC11320827

[CR42] Shrestha S, Koirala B, Dali M, Birajee G. Anterior Crowns in pediatric dentistry: a review. J Nepal Assoc Pediatr Dent. 2020;1(1):32–8.

[CR43] Soleimani F, Jalali H, Mostafavi AS, Zeighami S, Memarian M. Retention and clinical performance of zirconia crowns: a comprehensive review. Int J Dent. 2020;2020:1–6. 10.1155/2020/8846534 PMC7584951 33123199

[CR44] Sterne JAC, Savović J, Page MJ, Elbers RG, Blencowe NS, Boutron I, et al. RoB 2: a revised tool for assessing risk of bias in randomised trials. BMJ [internet]. 2019;7(6):4898. 10.1136/bmj.l4898. 10.1136/bmj.l4898 31462531

[CR45] Suresh B, Gunasekaran R. Evaluating the success of preformed resin bonded composite strip crowns for the primary central incisor in restoring carious primary canine teeth: a retrospective analysis. Cureus. 2024;16(8):e67012. 39280454 10.7759/cureus.67012 PMC11402461

[CR46] Vaghela LL, Patel MC, Bhatt RK, Patel CN, Joshi KR. Clinical performance and parental satisfaction with composite strip crown and prefabricated zirconia crown for primary anterior teeth: a randomized clinical trial. J Contemp Dent Pract. 2021;22(12):1462–70. 35656688

[CR47] Waggoner WF. Restoring primary anterior teeth. Pediatr Dent. 2002;24(5):511–6. 12412967

[CR500] Waggoner WF, Cohen H. Failure strength of four veneered primary stainless steel crowns. Pediatr Dent 1995;17(1):36–40. 7899100

[CR48] Walia T, Salami AA, Bashiri R, Hamoodi OM, Rashid F. A randomised controlled trial of three aesthetic full-coronal restorations in primary maxillary teeth. Eur J Paediatr Dent. 2014;15(2):113–8. 25102458

[CR49] Yanover L, Tickotsky N, Waggoner W, Kupietzky A, Moskovitz M. Zirconia crown performance in primary maxillary anterior teeth: a retrospective photographic and radiographic cohort study. Eur Arch Paediatr Dent. 2021a;22(3):417–23. 33029745 10.1007/s40368-020-00571-5

[CR50] Yanover L, Waggoner W, Kupietzky A, Moskovitz M, Tickotsky N. Parental and dentist satisfaction with primary anterior zirconia crowns: a case series analysis. Children. 2021b;8(6):451. 34073572 10.3390/children8060451 PMC8228435

[CR51] Zhang B, Wang J, Han X, Fang R, Wang Z, Hui Z, et al. Success rate of the treatment of early childhood caries under general anesthesia: a retrospective cohort study in different periods. Front Pediatr. 2023;11:1117935. 37063665 10.3389/fped.2023.1117935 PMC10102464

