# Supplementary material for: Prefabricated zirconia crowns versus alternative full-coverage restorations for primary anterior teeth: a systematic review of clinical outcomes
Source: Eur Arch Paediatr Dent. 2025 Dec 9;27(4):983–95. doi: 10.1007/s40368-025-01142-2 (PMC13461809; doi:10.1007/s40368-025-01142-2)
Supplement: Supplementary file 1 [file 40368_2025_1142_MOESM1_ESM.docx]

**Supplementary file 1: Search strategy performed for databases**

| **Database** | **Applied Search Strategy** |
| --- | --- |
| **PubMed**  August, 2025 | ("zirconia crown"[Title/Abstract] OR "zirconia crowns"[Title/Abstract] OR "prefabricated zirconia crown"[Title/Abstract] OR "prefabricated zirconia crowns"[Title/Abstract] OR "monolithic zirconia"[Title/Abstract] OR "EZ-Pedo"[Title/Abstract] OR "NuSmile"[Title/Abstract] OR "Kinder Krowns"[Title/Abstract] OR "Cheng crowns"[Title/Abstract] OR "Signature crowns"[Title/Abstract]) AND ("primary teeth"[MeSH Terms] OR "primary tooth"[Title/Abstract] OR "deciduous teeth"[Title/Abstract] OR "primary dentition"[Title/Abstract] OR "primary anterior teeth"[Title/Abstract] OR "maxillary primary incisors"[Title/Abstract]) AND ("child"[MeSH Terms] OR "children"[Title/Abstract] OR "pediatric"[Title/Abstract] OR "paediatric"[Title/Abstract] OR "pediatric dentistry"[MeSH Terms]) AND ("retention"[Title/Abstract] OR "crown survival"[Title/Abstract] OR "clinical performance"[Title/Abstract] OR "crown failure"[Title/Abstract] OR "esthetic"[Title/Abstract] OR "aesthetic"[Title/Abstract] OR "color match"[Title/Abstract] OR "gingival health"[Title/Abstract] OR "plaque accumulation"[Title/Abstract] OR "gingival index"[Title/Abstract] OR "secondary caries"[Title/Abstract] OR "enamel wear"[Title/Abstract] OR "opposing tooth wear"[Title/Abstract] OR "parental satisfaction"[Title/Abstract] OR "child satisfaction"[Title/Abstract]) |
| **Web of Science**  August, 2025 | TS=("zirconia crown" OR "zirconia crowns" OR "prefabricated zirconia crown" OR "monolithic zirconia" OR "EZ-Pedo" OR "NuSmile" OR "Kinder Krowns") AND TS=("primary teeth" OR "deciduous teeth" OR "anterior primary teeth" OR "primary dentition" OR "maxillary incisors") AND TS=("children" OR "pediatric dentistry" OR "paediatric dentistry") AND TS=("retention" OR "esthetic outcome" OR "gingival health" OR "plaque accumulation" OR "enamel wear" OR "recurrent caries" OR "parental satisfaction" OR "clinical performance") |
| **Scopus**  August, 2025 | TITLE-ABS-KEY("zirconia crowns" OR "prefabricated zirconia crowns" OR "monolithic zirconia" OR "EZ-Pedo" OR "NuSmile" OR "Kinder Krowns") AND TITLE-ABS-KEY("primary teeth" OR "primary dentition" OR "deciduous teeth" OR "anterior primary teeth" OR "maxillary primary incisors") AND TITLE-ABS-KEY("children" OR "child" OR "pediatric dentistry" OR "paediatric") AND TITLE-ABS-KEY("retention" OR "clinical performance" OR "esthetic outcomes" OR "aesthetic outcomes" OR "color match" OR "gingival index" OR "plaque accumulation" OR "enamel wear" OR "tooth wear" OR "satisfaction" OR "recurrent caries") |
| **Embase**  August, 2025 | ('zirconium oxide crown'/exp OR 'zirconia crown':ti,ab,kw OR 'prefabricated zirconia crown':ti,ab,kw OR 'EZ-Pedo':ti,ab,kw OR 'NuSmile':ti,ab,kw OR 'Kinder Krowns':ti,ab,kw OR 'monolithic zirconia':ti,ab,kw) AND ('primary tooth'/exp OR 'primary teeth':ti,ab,kw OR 'deciduous teeth':ti,ab,kw OR 'anterior primary teeth':ti,ab,kw OR 'primary dentition':ti,ab,kw) AND ('child'/exp OR 'children':ti,ab,kw OR 'pediatric dentistry'/exp OR 'paediatric':ti,ab,kw) AND ('clinical performance':ti,ab,kw OR 'crown retention':ti,ab,kw OR 'esthetic outcome':ti,ab,kw OR 'aesthetic outcome':ti,ab,kw OR 'plaque index':ti,ab,kw OR 'gingival index':ti,ab,kw OR 'enamel wear':ti,ab,kw OR 'tooth wear':ti,ab,kw OR 'treatment satisfaction':ti,ab,kw OR 'parent satisfaction':ti,ab,kw) |
| **Cochrane Library**  August, 2025 | # (zirconia crowns OR prefabricated zirconia crowns OR monolithic zirconia OR EZ-Pedo OR NuSmile OR Kinder Krowns) AND (primary teeth OR primary tooth OR deciduous teeth OR primary dentition OR anterior primary teeth OR maxillary primary incisors) AND (children OR pediatric dentistry OR paediatric) AND (retention OR clinical success OR esthetics OR gingival health OR plaque index OR enamel wear OR parental satisfaction OR child satisfaction OR survival rate) |
