# Supplementary material for: Prefabricated zirconia crowns versus alternative full-coverage restorations for primary anterior teeth: a systematic review of clinical outcomes
Source: Eur Arch Paediatr Dent. 2025 Dec 9;27(4):983–95. doi: 10.1007/s40368-025-01142-2 (PMC13461809; doi:10.1007/s40368-025-01142-2)
Supplement: Supplementary file 2 [file 40368_2025_1142_MOESM2_ESM.docx]

**Supplementary file 2: List of excluded studies and their reasons for exclusion**

| **Study** | **Reason for Exclusion** | **Reference** |
| --- | --- | --- |
| Lin et al. 2021 | Did not evaluate 3D-printed crowns | Lin, B., Khatri, A., & Hong, M. 2021. Comparison of fracture strengths among different commonly placed anterior esthetic restorations for primary dentition: An in vitro study. *The Journal of Clinical Pediatric Dentistry*, 45(3), 171–176. <https://doi.org/10.17796/1053-4625-45.3.5> |
| El Hayek et al. 2022 | Did not evaluate 3D-printed crowns | El Hayek, J., El Osta, N., & Farhat Mchayleh, N. 2022. Fracture strength of preformed zirconia crown and new custom-made zirconia crown for the restoration of deciduous molars: an in vitro study. *European Archives of Paediatric Dentistry*, 23(4), 333–339. <https://doi.org/10.1007/s40368-022-00691-0> |
| Prabhu et al. 2022 | Did not evaluate 3D-printed crowns | Prabhu, D., Anantharaj, A., Praveen, P., Rani, S. P., & Sudhir, R. 2022. A clinical and radiographic comparative evaluation of custom-made zirconia crowns using CAD-CAM and stainless steel crowns in primary molars. *Journal of Indian Society of Pedodontics and Preventive Dentistry*, 40(1), 34–42. <https://doi.org/10.4103/jisppd.jisppd_269_21> |
| Aydin & Uguz 2023 | Full text not accessible | Aydin, N., & Uguz, H. N. 2023. Shear bond strength of permanent 3D-printed resin and milled zirconia to primary teeth using different luting agents. *American Journal of Dentistry*, 36(5), 239–245. PMID: 37865811 |
| Refaie et al. 2023 | Focused on permanent teeth, not primary teeth | Refaie, A., Bourauel, C., Fouda, A. M., Keilig, L., & Singer, L. 2023. The effect of cyclic loading on the fracture resistance of 3D-printed and CAD/CAM milled zirconia crowns—an in vitro study. *Clinical Oral Investigations*, 27(10), 6125–6133. <https://doi.org/10.1007/s00784-023-05229-2> |
| El Hayek et al. 2024 | Did not evaluate 3D-printed crowns | El Hayek, J. E., Tohme, H., Nasr, L., El Hachem, R., Kabbani, N., & Farhat Mchayleh, N. 2024. Fracture strength of preformed zirconia crown and CAD–CAM zirconia, ceramic, and hybrid composite crowns for the restoration of primary molars: An in vitro study. *International Journal of Paediatric Dentistry*, 34(6), 721–728. <https://doi.org/10.1111/ipd.13171> |
| Matha et al., 2024 | Did not evaluate 3D-printed crowns | Matha, N., Kumar, K.S., Reddy, B.V.T., et al. 2024. Comparative Evaluation of Polymethyl Methacrylate and Thermoforming Crowns as Semipermanent Crowns in Primary Molars: An In Vivo Study. *International Journal of Clinical Pediatric Dentistry*, 17(S-1), S43–S54. https://doi.org/10.5005/jp-journals-10005-2815 |
| Park et al. 2024 | Focused on permanent teeth, not primary teeth | Park, Y., Kim, J., Kang, Y.-J., Shim, E.-Y., & Kim, J.-H. 2024. Comparison of Fracture Strength of Milled and 3D-Printed Crown Materials According to Occlusal Thickness. *Materials*, 17, 4645. <https://doi.org/10.3390/ma17184645> |
| Bani-Hani et al. 2025 | Focused on permanent teeth, not primary teeth | Bani-Hani, T., Al-Fodeh, R. S., Al-Wahadni, A. M., Abu-Alhaija, E. S., & Al-Hakam, M. 2025. An in-vitro investigation into the fracture resistance of prefabricated and custom-made zirconia crowns for permanent molars in children. *Dentistry Journal*, 13(2), 64. <https://doi.org/10.3390/dj13020064> |
| Gul et al. 2025 | Focused on permanent teeth, not primary teeth | Gul, B. C., Demirci, F., Baki, N., Bahce, E., & Özcan, M. 2025. Mechanical analysis of 3D printed dental restorations manufactured using different resins and validation with FEM analysis. *BMC Oral Health*, 25, 131. <https://doi.org/10.1186/s12903-024-05384-2> |
| Salman et al. 2025 | Did not evaluate 3D-printed crowns | Salman, N. R., Khattab, N. M. A., Gomaa, Y., & Elheeny, A. A. H. 2025. In vitro comparison of marginal fit between CAD/CAM hybrid ceramic crowns and prefabricated zirconia crowns for primary molars. *Odontology*. <https://doi.org/10.1007/s10266-025-01089-2> |
| Zhai et al. 2025 | Focused on permanent teeth, not primary teeth | Zhai, Z., Qian, C., Jiao, T., & Sun, J. 2025. In vitro fracture and fatigue resistance of monolithic zirconia crowns fabricated by stereolithography. *The Journal of Prosthetic Dentistry*, 133(2), 584–591. <https://doi.org/10.1016/j.prosdent.2023.02.017> |
| Zhu et al. 2025 | Focused on permanent teeth, not primary teeth | Zhu, H., Zhou, Y., Jiang, J., Wang, Y., & He, F. 2025. Accuracy and margin quality of advanced 3D printed monolithic zirconia crowns. *The Journal of Prosthetic Dentistry*, 133, 1284–1292. <https://doi.org/10.1016/j.prosdent.2023.07.004> |
