# Supplementary material for: Prefabricated zirconia crowns versus alternative full-coverage restorations for primary anterior teeth: a systematic review of clinical outcomes
Source: Eur Arch Paediatr Dent. 2025 Dec 9;27(4):983–95. doi: 10.1007/s40368-025-01142-2 (PMC13461809; doi:10.1007/s40368-025-01142-2)
Supplement: Supplementary file 3 [file 40368_2025_1142_MOESM3_ESM.docx]

**Supplementary file 3: Quality assessment for randomized clinical trials**

Risk of Bias 2 (RoB 2) Assessment for Gill et al. [1]

| **Domain** | **Signaling Question** | **Response** | **Justification** | **Direct Reference from Study** | **Judgment** |
| --- | --- | --- | --- | --- | --- |
| **Randomization** | Was the allocation sequence random? | Yes | Randomization sequence created using statistical software. | "The randomization sequence was created using Stata 12.0 software (StataCorp, College Station, Texas) using random block sizes." (Methods section) | **Some concerns** |
|  | Was the allocation sequence concealed? | Probably yes | Block randomization implemented; concealment method details not explicitly reported. | "Random block sizes, which were known only to the study statistician." (Methods section) |  |
|  | Any baseline differences? | Yes | Significant differences in gender, insurance type, and dmft at baseline. | "Although the children were randomized to groups, gender, insurance type, and dmft at PAC placement were significantly different among the three PAC types." (Results section) |  |
| **Deviations from intended interventions** | Were participants aware of assignment? | Probably no | Single-blind with children under anesthesia, likely preventing participant awareness. | "Single-blinded, randomized controlled clinical trial." (Methods section) | **Some concerns** |
|  | Were carers aware of assignment? | Probably yes | Operators inherently knew crown type used. | "Four pediatric dentists were calibrated on tooth preparation and crown placement." (Methods section) |  |
|  | Was appropriate analysis used? | Yes | Outcomes analyzed according to randomization. | "The tooth was the unit of analysis; although the study was randomized at the patient level, all analysis was performed at the tooth level." (Methods section) |  |
| **Missing outcome data** | Data for all/most participants? | Yes | Minimal dropout, clearly accounted for. | "Forty-seven children (135 teeth) were enrolled... At 12 months, crown retention was significantly lower for CSCs." (Results section) | **Low risk** |
| **Measurement of outcomes** | Method of measurement inappropriate? | No | Validated and standardized assessment tools used. | "An evaluation rating system similar to the USPHS Ryge Criteria." (Methods section) | **Low risk** |
|  | Were assessors aware of assignment? | No | Assessors were calibrated and blinded. | "Demographic and tooth-related variables assessed by calibrated examiners." (Methods section) |  |
|  | Could assessment be influenced? | No | Objective measurement methods used. | "Crowns photographed at baseline and again at recall visits." (Methods section) |  |
| **Selection of reported result** | Analysis plan pre-specified? | No information | No statistical analysis plan or trial registration details provided. | None provided | **Some concerns** |
|  | Multiple outcome measures in domain? | No | Outcomes predefined and consistently reported. | "Clinical outcomes at 12 months were evaluated." (Methods section) |  |
|  | Multiple analyses? | No | Single predefined outcome analysis reported. | "All data entered into REDCap and analyzed in Stata 15.1." (Methods section) |  |

Overall Risk of Bias Judgment: Some concerns

RoB 2 assessment for Alaki et al. [2]

| **Domain** | **Signaling Question** | **Response** | **Justification** | **Direct Reference from Study** | **Judgment** |
| --- | --- | --- | --- | --- | --- |
| **Randomization** | Was the allocation sequence random? | Yes | Randomization done using statistical software (SPSS). | "Randomization was done using SPSS software version 20.0 (IBM Corp., Armonk, NY)." (Methods section) | **Low risk** |
|  | Was the allocation sequence concealed? | Yes | Sealed, opaque envelopes were used to conceal assignment. | "Allocation concealment was assured by handling sequentially numbered opaque sealed envelopes..." (Methods section) |  |
|  | Any baseline differences? | No | Demographic data presented; no significant differences between groups noted. | "Full coronal restorations were placed on 120 primary maxillary anterior teeth... no drop out until the 12 months follow up." (Results section) |  |
| **Deviations from intended interventions** | Were participants aware of assignment? | Probably no | All procedures performed under nitrous oxide sedation; children unlikely to be aware. | "All treatments were done under nitrous oxide sedation..." (Procedure section) | **Low risk** |
|  | Were carers aware of assignment? | Yes | Operators needed to apply specific protocols per crown type. | "The same examiner was responsible of preparation and evaluation." (Examiner calibration section) |  |
|  | Was appropriate analysis used? | Yes | Statistical methods clearly described; outcomes analyzed by treatment assignment. | "Simple descriptive statistics were applied... Friedman analysis was used..." (Statistical analysis section) |  |
| **Missing outcome data** | Data for all/most participants? | Yes | No dropouts reported; full follow-up achieved at 12 months. | "No drop out until the 12 months follow up." (Results section) | **Low risk** |
| **Measurement of outcomes** | Method of measurement inappropriate? | No | Validated scales and indexes used to assess outcomes. | "Silness and Löe criteria... USPHS Alpha criteria... Smith and Knight Tooth Wear Index." (Table 1) | **Low risk** |
|  | Were assessors aware of assignment? | Probably yes | Same examiner prepared and evaluated restorations. | "The same examiner was responsible of preparation and evaluation." (Examiner calibration section) |  |
|  | Could assessment be influenced? | Probably no | Objective, standardized indices were applied; intra-examiner reliability was excellent. | "The clinical calibration results produced by the examiner were found to be at an excellent agreement range (0.97)." (Results section) |  |
| **Selection of reported result** | Analysis plan pre-specified? | No information | No mention of pre-specified analysis plan or trial registration timing. | "The presented research was registered retrospectively... on 6th of August 2017..." (Methods section) | **Some concerns** |
|  | Multiple outcome measures in domain? | No | Outcomes were clearly defined and consistently reported. | "The parameters that were evaluated in this study are listed in Table 1." (Methods section) |  |
|  | Multiple analyses? | No | Each outcome was analyzed once; results clearly presented per time point. | Tables 3–7 (Results section) |  |

Overall Risk of Bias Judgment: Some concerns

RoB 2 Assessment for Vaghela et al. [3]

| **Domain** | **Signaling Question** | **Response** | **Justification** | **Direct Reference from Study** | **Judgment** |
| --- | --- | --- | --- | --- | --- |
| **Randomization** | Was the allocation sequence random? | Yes | Computer-generated randomization was used. | "Randomly allocated based on computer randomization into one of the two groups..." (Materials and Methods) | **Some concerns** |
| **Was the allocation sequence concealed?** | No information | The method of allocation concealment is not described. | Not mentioned in text |  |  |
| **Any baseline differences?** | No | No statistically significant baseline imbalances reported. | No differences mentioned between groups at baseline. |  |  |
| **Deviations from intended interventions** | Were participants aware of assignment? | Probably no | Children aged 3–6 years were unlikely to understand assignment, and no mention of informing participants. | "Children aged 3–6 years"; no mention of awareness | **Some concerns** |
| **Were carers aware of assignment?** | Yes | Operators inherently knew the type of crown used. | Crown preparation and cementation procedures differ and were carried out by the operators. |  |  |
| **Was appropriate analysis used?** | Yes | Groups analyzed as randomized and appropriate statistical tests applied. | "Statistical analysis was done using Chi-square test (p <0.05)." (Materials and Methods) |  |  |
| **Missing outcome data** | Data for all/most participants? | Yes | Minimal dropout—only one dropout per group. | "One child in group A and one child in group B failed to come for the follow-up at 9 months." (Results) | **Low risk** |
| **Measurement of outcomes** | Method of measurement inappropriate? | No | Validated and standardized criteria used. | "Modified United States Public Health Service (USPHS) criteria" and "5-point Likert scale" used. (Materials and Methods) | **Low risk** |
| **Were assessors aware of assignment?** | No | Outcomes were objectively measured and assessed using standardized criteria. | Objective tools (USPHS, Likert) likely minimize bias, though explicit blinding not mentioned. |  |  |
| **Could assessment be influenced?** | No | Most outcomes were clinical and parental ratings, reducing risk of detection bias. | Objective outcomes with scoring systems applied. |  |  |
| **Selection of reported result** | Analysis plan pre-specified? | No information | No protocol or registration referenced. | Not reported | **Some concerns** |
| **Multiple outcome measures in domain?** | No | Outcomes were predefined and consistently reported. | "Clinical success and parental satisfaction" evaluated with specific criteria. |  |  |
| **Multiple analyses?** | No | Single analysis per outcome reported. | "Statistical analysis was done using Chi-square test (p <0.05)." |  |  |

Overall Risk of Bias Judgment: Some concerns

RoB 2 Assessment for Misgar et al. [4]

| **Domain** | **Signaling Question** | **Response** | **Justification** | **Direct Reference from Study** | **Judgment** |
| --- | --- | --- | --- | --- | --- |
| **Randomization** | Was the allocation sequence random? | No information | The study stated that participants were randomly divided, but no method of sequence generation was described. | "The crowns were randomly divided into 3 groups with 10 teeth falling in each group." (Materials & Methods) | **Some concerns** |
|  | Was the allocation sequence concealed? | No information | The method for allocation concealment was not reported. | Not reported |  |
|  | Any baseline differences? | No | No baseline differences were mentioned or compared between groups. | Not mentioned |  |
| **Deviations from intended interventions** | Were participants aware of assignment? | Probably no | Children were aged 4–9 and were likely unaware of the intervention assigned. No statement indicated they were informed. | Not explicitly stated, but inferred from age and setting | **Some concerns** |
|  | Were carers aware of assignment? | Yes | Operators must have been aware due to differences in crown materials and preparation techniques. | "Zirconia, luxa, and strip crowns were placed using different techniques." (Materials & Methods) |  |
|  | Was appropriate analysis used? | Yes | Data were analyzed according to randomized crown groups. | "Data was collected and result was analysed using chi-square test and SPSS software." (Materials & Methods) |  |
| **Missing outcome data** | Data for all/most participants? | Yes | No dropouts were reported, and all 30 crowns were followed for the full 9 months. | "A total of 30 deciduous crowns were included." (Results section) | **Low risk** |
| **Measurement of outcomes** | Method of measurement inappropriate? | No | Gingival health was measured using a WHO probe, and caries were assessed clinically. | "Gingival health was measured using WHO probe..." (Results section) | **Low risk** |
|  | Were assessors aware of assignment? | No information | No detail was given about blinding of outcome assessors. | Not reported |  |
|  | Could assessment be influenced? | Probably yes | The outcome (bleeding and caries) could be influenced by knowledge of the crown type. | Not reported |  |
| **Selection of reported result** | Analysis plan pre-specified? | No information | There was no mention of a trial registration or analysis plan. | Not reported | **Some concerns** |
|  | Multiple outcome measures in domain? | No | Outcomes (bleeding on probing and secondary caries) were predefined and consistently reported. | "Patients were recalled after 3,6 and 9 months to evaluate the gingival health and secondary caries." (Materials & Methods) |  |
|  | Multiple analyses? | No | A single method of statistical analysis was used. | "Result was analysed using chi-square test and SPSS software." (Materials & Methods) |  |

Overall Risk of Bias Judgment: Some concerns

RoB 2 Assessment for Özdemir et al. [5]

| **Domain** | **Signaling Question** | **Response** | **Justification** | **Direct Reference from Study** | **Judgment** |
| --- | --- | --- | --- | --- | --- |
| **Randomization** | Was the allocation sequence random? | Yes | Randomization was done using a contingency number table from random.org. | “The randomization was done using a contingency number table from www.random.org.” (Methods) |  |
|  | Was the allocation sequence concealed? | Probably yes | Sequentially numbered sealed envelopes were used, but no details on opacity or tamper-resistance. | “The results were kept in sequentially numbered sealed envelopes.” (Methods) |  |
|  | Any baseline differences? | No | No significant baseline imbalances reported or discussed. | Not explicitly stated but groups appeared comparable. | **Some concerns** |
| **Deviations from intended interventions** | Were participants aware of assignment? | Probably yes | Blinding not possible due to visible differences in crown types. | “Blinding of the operator was not possible due to the distinct physical and procedural properties of crowns.” (Methods) |  |
|  | Were carers aware of assignment? | Yes | Operators could not be blinded; patients were treated under local anesthesia. | “All treatments were performed by an experienced, calibrated pediatric dentist.” (Methods) |  |
|  | Was appropriate analysis used? | Yes | All randomized teeth analyzed per protocol. | “The teeth were randomized... Patients’ oral hygiene and clinical variables... were assessed.” (Abstract & Methods) | **Some concerns** |
| **Missing outcome data** | Data for all/most participants? | Yes | Dropouts (23 teeth) and exfoliations (14 teeth) were clearly reported and excluded appropriately. | “During the 18-month follow-up period, 23 teeth were lost... and 14 teeth were exfoliated.” (Results) | **Low risk** |
| **Measurement of outcomes** | Method of measurement inappropriate? | No | Validated indices and calibrated assessors used. | “Silness and Löe plaque index... Löe and Silness gingival index...” (Methods) |  |
|  | Were assessors aware of assignment? | Probably no | Two independent, calibrated researchers assessed outcomes. | “All clinical assessments were performed independently by two calibrated researchers.” (Methods) |  |
|  | Could assessment be influenced? | No | Calibration and consensus ensured objectivity. | “In case of disagreement, a consensus assessment was reached.” (Methods) | **Low risk** |
| **Selection of reported result** | Analysis plan pre-specified? | No information | Trial registration ID mentioned but no SAP or protocol available. | “The study protocol was registered on ClinicalTrials.gov database (NCT05034003).” (Methods) |  |
|  | Multiple outcome measures in domain? | No | Predefined outcomes consistently reported. | “Clinical outcomes... included gingival health, plaque... retention, color match...” (Abstract) |  |
|  | Multiple analyses? | No | Single predefined outcome set analyzed. | “Data were statistically analyzed... For all tests, the level of significance was set at P<0.05.” (Methods) | **Some concerns** |

Overall Risk of Bias Judgment: Some concerns

RoB 2 Assessment for Ravada et al. [6]

| **Domain** | **Signaling Question** | **Response** | **Justification** | **Direct Reference from Study** | **Judgment** |
| --- | --- | --- | --- | --- | --- |
| Randomization | Was the allocation sequence random? | No information | The study mentions participants were "randomly divided," but no method or tool is described. | “The subjects were randomly divided to two groups…” (Methods) |  |
|  | Was the allocation sequence concealed? | No information | No details provided on how allocation was concealed from the investigators or participants. | Not reported |  |
|  | Any baseline differences? | No | No baseline imbalances were reported; the groups appear comparable. | No mention of baseline differences across groups. | **Some concerns** |
| Deviations from intended interventions | Were participants aware of assignment? | Probably yes | No mention of blinding. Participants and/or guardians were likely aware of crown type. | Not reported |  |
|  | Were carers aware of assignment? | Yes | Operators applying different types of crowns must have known which was used. | “The restorations were done according to the treatment protocols and the manufacturer’s guidelines.” (Methods) |  |
|  | Was appropriate analysis used? | Yes | Outcomes analyzed based on randomized groups; no per-protocol switching described. | “These readings were compared using Chi square test…” (Methods) | **Some concerns** |
| Missing outcome data | Data for all/most participants? | Yes | The study followed all patients for 12 months and accounted for loss/failure events clearly. | “...two crowns were lost… failure was more in the strip crown group…” (Results) | **Low risk** |
| Measurement of outcomes | Method of measurement inappropriate? | No | Valid outcome measures: plaque, gingival bleeding, failure, wear, etc., measured clinically. | Parameters like gingival health and plaque index assessed at 3, 6, 12 months. |  |
|  | Were assessors aware of assignment? | No information | There is no mention of blinding of assessors or calibration. | Not reported |  |
|  | Could assessment be influenced? | Probably yes | Outcome measures could be influenced due to visual inspection and no blinding. | “...clinically observed for any sign of abrasion…” (Results) | **Some concerns** |
| Selection of reported result | Analysis plan pre-specified? | No information | No mention of trial registration or protocol. | Not reported |  |
|  | Multiple outcome measures in domain? | No | The outcomes were predefined and consistently reported. | Gingival health, caries, failure, and wear reported at 3, 6, and 12 months. |  |
|  | Multiple analyses? | No | Single statistical approach reported. | “Chi square test… p<0.05” (Methods) | **Some concerns** |

Overall Risk of Bias Judgment: High Risk

RoB 2 Assessment for Labbé et al. [7]

| **Domain** | **Signaling Question** | **Response** | **Justification** | **Direct Reference from Study** | **Judgment** |
| --- | --- | --- | --- | --- | --- |
| Randomization | Was the allocation sequence random? | Yes | Random number sequence generated using Microsoft Excel 2018. | "Participant group assignment was revealed from a computer-generated simple random number sequence with a one-to-one allocation ratio (Microsoft® Excel 2018...)" (Methods section) |  |
| Randomization | Was the allocation sequence concealed? | Yes | Sequence was concealed until immediately before treatment. | "The randomization sequence was concealed until immediately prior to treatment of the incisors." (Methods section) |  |
| Randomization | Any baseline differences? | No | Demographic and tooth-level factors showed no statistical differences. | "Chi-square tests demonstrated no differences between groups for all factors evaluated." (Table 1 footnote) | **Low risk** |
| Deviations from intended interventions | Were participants aware of assignment? | No | Treatment done under general anesthesia; children unlikely to be aware. | Implicitly indicated: children were treated under general anesthesia, no indication of awareness. |  |
| Deviations from intended interventions | Were carers aware of assignment? | Probably yes | Operators likely knew which material was used due to procedural differences. | Not explicitly stated, but preparation protocols differ significantly between SC and ZC groups. | **Some concerns** |
| Deviations from intended interventions | Was appropriate analysis used? | Yes | Used generalized estimating equation (GEE) to account for clustering by incisors. | "A generalized estimating equation (GEE) was used to account for the lack of independence among incisors." (Results section) |  |
| Missing outcome data | Data for all/most participants? | Yes | Follow-up at 6 and 12 months with minimal missing data. | "Participants were appointed for clinical follow-up at six and 12 months..." (Methods section) | **Low risk** |
| Measurement of outcomes | Method of measurement inappropriate? | No | Validated outcome assessment system using blinded evaluators and standard photographs. | "Standardized photographs were acquired at each appointment and reviewed by two disinterested pediatric dentists." (Methods section) |  |
| Measurement of outcomes | Were assessors aware of assignment? | No | Blinded outcome assessment by independent raters. | "The raters were blinded to all participants’ identifying information." (Methods section) |  |
| Measurement of outcomes | Could assessment be influenced? | No | Objective visual criteria applied on photos; blinded reviewers. | "...using a random number generator... photographs were reviewed on a MacBook Pro... blinding maintained." (Methods section) | **Low risk** |
| Selection of reported result | Analysis plan pre-specified? | Yes | Study registered at ClinicalTrials.gov; analysis methods described. | "The investigation was registered with ClinicalTrials.gov (ID NCT03889535)." (Methods section) |  |
| Selection of reported result | Multiple outcome measures in domain? | No | Primary outcome (restoration status) predefined and used consistently. | "Each incisor was rated as intact (I), damaged (D), or requiring treatment (TR)..." (Methods section) |  |
| Selection of reported result | Multiple analyses? | No | GEE analysis performed as planned; no multiple or selective analyses reported. | "...exported into an Excel spreadsheet... statistical analysis." (Methods section) | **Low risk** |

Overall Risk of Bias Judgment: Some concerns

**References**

1.	Gill A, Garcia M, Won An S, Scott J, Seminario AL. Clinical Comparison of Three Esthetic Full-Coverage Restorations in Primary Maxillary Incisors at 12 Months. Pediatr Dent. 2020;42:367–72. http://www.ncbi.nlm.nih.gov/pubmed/33087221

2.	Alaki SM, Abdulhadi BS, AbdElBaki MA, Alamoudi NM. Comparing zirconia to anterior strip crowns in primary anterior teeth in children: a randomized clinical trial. BMC Oral Health. 2020;20:1–11. 10.1186/s12903-020-01305-1

3.	Vaghela LL, Patel MC, Bhatt RK, Patel CN, Joshi KR. Clinical Performance and Parental Satisfaction with Composite Strip Crown and Prefabricated Zirconia Crown for Primary Anterior Teeth: A Randomized Clinical Trial. J Contemp Dent Pract. 2021;22:1462–70. 10.5005/jp-journals-10024-3264

4.	Misgar BA, Goyal V, Rani N. Comparative evaluation between zirconia, luxa andstrip crowns: A randomised controlled trial. J Adv Med Dent Sci Res. 2022;22:1462–70. 10.5005/jp-journals-10024-3264

5.	Ozdemir M, Unverdi GE, Geduk N, Ballikaya E, Cehreli ZC. Clinical Comparison of Preformed Zirconia and Composite Strip Crowns in Primary Maxillary Incisors: 18-Month Results of a Prospective, Randomized Trial. Pediatr Dent. 2022;44:416–22. http://www.ncbi.nlm.nih.gov/pubmed/36947757

6.	Ravada SUS, Nara A, Gavarraju DN, Arya J, Kapur I, Begani S. Comparison of efficiency of different crowns in primary children an original research. Int J Health Sci (Qassim). 2022;6:13197–202. 10.53730/ijhs.v6nS1.8472

7.	Labbé S, Garisto GA, Barrett EJ, Casas MJ. A Comparison of Primary Maxillary Incisor Zirconia and Composite Resin Strip Crowns: A One-year Feasibility Study. Pediatr Dent. 2023;45:113–6. http://www.ncbi.nlm.nih.gov/pubmed/37106539
